# Arterial stiffness is associated with cancer mortality: Insight from Kailuan study

**DOI:** 10.1002/cam4.6251

**Published:** 2023-06-23

**Authors:** Jiatian Li, Tesfaldet Habtemariam Hidru, Yajuan Lin, Xinying Wang, Li Lin, Shuohua Chen, Yunlong Xia, Xiaolei Yang, Shouling Wu

**Affiliations:** ^1^ Department of Cardiology, Institute of Cardiovascular Diseases First Affiliated Hospital of Dalian Medical University Dalian China; ^2^ Health Department of Kailuan Group Tangshan China; ^3^ Department of Cardiology, Kailuan General Hospital North China University of Science and Technology Tangshan China

**Keywords:** arterial stiffness, cancer, malignancy, mortality

## Abstract

**Background:**

There is limited evidence on the association between arterial stenosis and the risk of all‐cause mortality in cancer patients (ACMC). This study investigated whether the status of arterial function and structure measured by brachial–ankle pulse wave velocity (baPWV) is associated with ACMC.

**Methods:**

A total of 43,943 Chinese adults underwent a baPWV examination. Cox proportional hazards model was used to assess the association between the baPWV values and ACMC.

**Results:**

During a total follow‐up duration of 3.81 ± 2.50 years, there were 157 deaths among 553 cancer cases diagnosed during the follow‐up. Patients with baPWV ≥18 m/s showed an increased risk of ACMC compared to patients with ideal vascular function. In the multivariate‐adjusted model, we observed a significant association between arterial stiffness severity and ACMC with a hazard ratio (HR) 2.72 (95% confidence interval [CI]: 1.55–4.80; *p* < 0.001) in those with baPWV ≥18 m/s. With a 1‐SD increase in baPWV, the HR (95% CI) for ACMC in the entire cohort, men, and patients ≤60 years old were 1.20 (95% CI: 1.03–1.41; *p* < 0.05), 1.20 (95% CI: 1.01–1.43; *p* < 0.05), and 1.44 (95% CI: 1.10–1.44; *p* = 0.008), respectively.

**Conclusions:**

Increased arterial stiffness measured by baPWV is associated with ACMC. The association between high baPWV (≥18 m/s) and risk of all‐cause mortality was prominent in men and those ≤60 years of age.

## 1 INTRODUCTION

Cancer is a leading cause of death worldwide.^1^, ^2^ What is more concerning, the burden of cancer is expected to increase globally.^3^ In parallel, the crude incidence and mortality rates of cancer have progressively risen since 2000 in China.^4^ According to the most recent epidemiological and statistical information, China is expected to have 3,210,000 cancer deaths and 4,820,000 new cases of malignancy in 2022.^5^ The morbidity and mortality of malignancy contribute to a disastrous socioeconomic burden. Therefore, there is an urgent need to discover risk factors influencing cancer mortality initiation and progression.

Arterial stiffness is a well‐established indicator of the rigidity of the artery wall.^6^ It can be determined by a simple and noninvasive procedure known as the brachial–ankle pulse wave velocity (baPWV). Of note, baPWV is defined as the distance between the brachial located 2–3 cm above the transverse line of the elbow fossa and the tibial artery located 1–2 cm above the superior lateral aspect of the medial malleolus divided by the pulse transit time between these two arteries.^7^ The relationship between arterial stiffness and deaths in people with Type 1 diabetes^8^ and hypertension (HTN) has been investigated in the past.^9^ Similarly, a community‐based study showed that arterial stiffness was positively associated with all‐cause mortality in elderly Japanese individuals.^10^ A lengthy, extensive, multicenter prospective observational cohort study examined baPWV in patients with Type II diabetes mellitus (DM) for its ability to predict overall and cause‐specific death rates and found that DM patients at the highest quartile of baPWV had a significantly greater risk of dying from cancer,^11^ suggesting that it may be worth investigating in cancer cohorts.

Recently, Parr et al. revealed that pulse pressure, an index of arterial stiffness, predicts cardiovascular disease (CVD) mortality in cancer patients <65 years.^12^ Their analysis showed that cancer patients with 60–70 mmHg pulse pressures had a higher risk of cardiovascular mortality than pulse pressure <50 mmHg. We extended their study based on baPWV, a different indicator of arterial stiffness than pulse pressure. PWV is considered the golden standard index of arterial stiffness,^13^ and baPWV is a simple and convenient measurement to be performed in large‐scale populations. Therefore, this study aimed to investigate the association between arterial stiffness status measured by baPWV and all‐cause mortality in cancer patients (ACMC).

## 2 METHODS

### 2.1 Study design and population

The Kailuan study (clinical trial number ChiCTR‐TNRC‐11001489) was a large prospective cohort investigation based on health monitoring data from a community population in Kailuan, Tangshan, Hebei, China. The Kailuan study was assessed with questionnaires and clinical and laboratory tests every 2 years. After receiving written informed consent from the participants, the clinical and laboratory data were collected for analysis. The Kailuan cohort study specifics have been documented.^14^ Arterial stiffness was evaluated from 2010 to 2016 using baPWV. Our study included participants in the Kailuan study who completed their first baPWV test between January 1, 2010 and December 31, 2018. Initially, 46,399 subjects were recruited.

**FIGURE 1:**
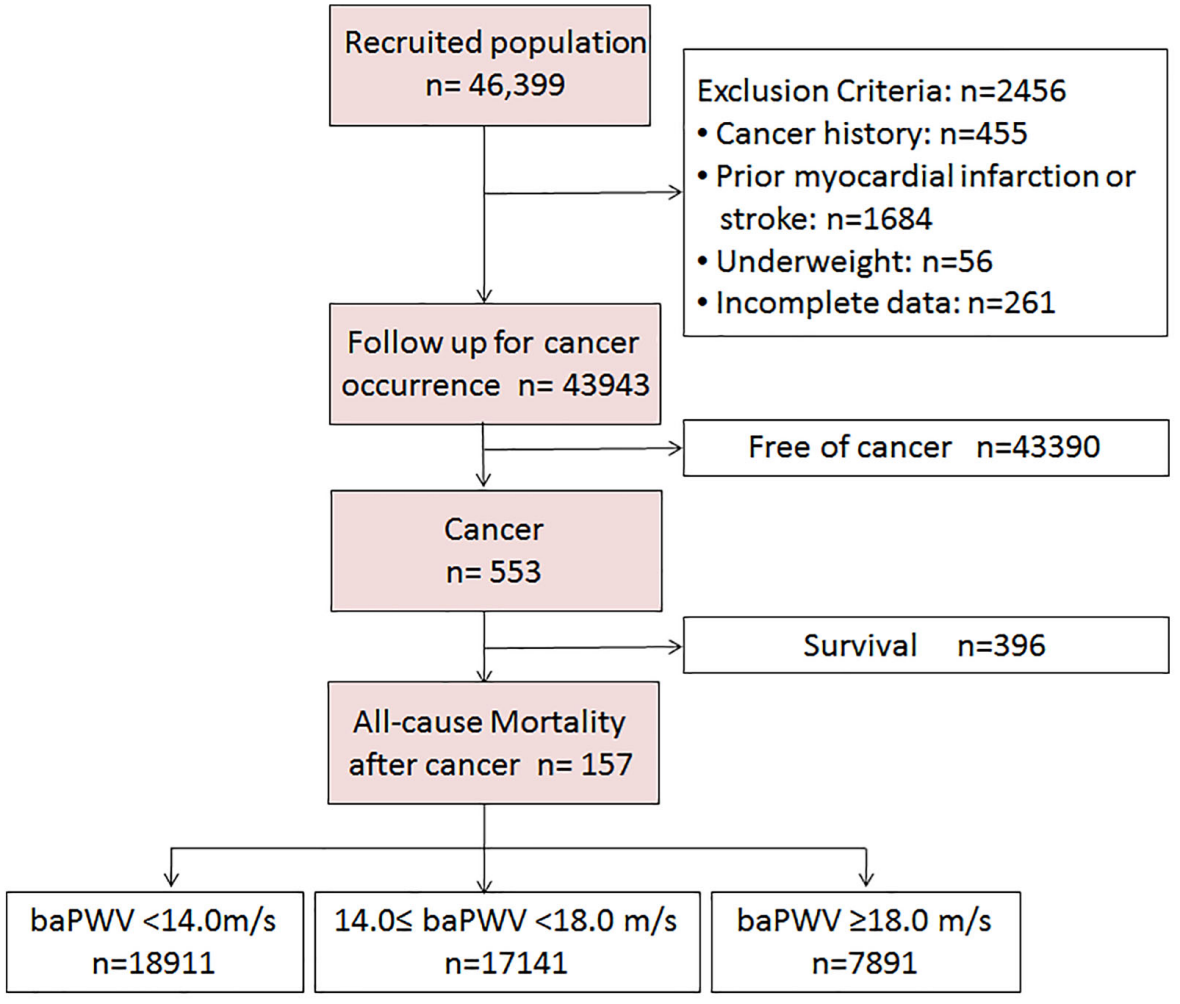
Flowchart of participants.

Further, we excluded those who had a history of cancer (*n* = 455), were diagnosed with serious cardiovascular and cerebrovascular diseases (*n* = 1684), had incomplete data (*n* = 261), and had underweight (*n* = 56). Then, 43,943 patients were further followed up for all‐cause mortality after cancer. During the follow‐up, there were 553 new cancer cases, and they were followed up for all causes of mortality. Figure 1 shows a brief description of the above process.

All‐cause mortality after the diagnosis of cancer was taken as the endpoint event. The endpoint event information was obtained annually through the Kailuan Social Security information system, and the endpoint events of the observation were recorded by trained medical personnel. A professional physician confirmed all diagnoses based on the hospital records. Each participant signed a written statement to participate in the study and consented to the use of the information for analysis. The Kailuan General Hospital Ethics Committee authorized the investigation, ensuring the study procedure complied with the Declaration of Helsinki II requirements.

### 2.2 Other variables

Each health assessment included the distribution of self‐administration questionnaires to gather information on anthropometry, lifestyle, previous medical history, and patient history of cancer. Weight (kg)/height^2^ (meter^2^) was applied to calculate the body mass index (BMI). After the participants sat for 30 minutes, the right arm was used to measure systolic blood pressure (SBP) and diastolic blood pressure (DBP) three times, and the average reading of the three blood pressure measurements were documented as the SBP and DBP readings. In this study, SBP ≥140 mmHg or DBP ≥90 mmHg, self‐reported history of HTN, or current use of antihypertensive medication were all considered HTN. The formula for determining mean arterial pressure (MAP) was (SBP + 2DBP)/3. Participants were advised to refrain from eating, smoking, and drinking for at least 8 h before a general medical checkup and blood and urine specimen collection. Biochemical analysis was carried out to assess fasting blood glucose (FBG) and high‐sensitivity C‐reactive protein (hs‐CRP) levels. FBG ≥7 mmol/L or self‐reported usage of glucose‐lowering medications indicated DM. A basic enzymatic approach was used to measure blood total cholesterol (TC) and high‐density lipoprotein (HDL) levels. The Chronic Kidney Disease Epidemiology Collaboration method was used to detect the estimated glomerular filtration rate (eGFR) from creatinine. Weight less than 30 kg was deemed underweight. Employing an enzyme‐linked immunosorbent assay (ELISA), the hepatitis B surface antigen (HBsAg) in venous blood was identified, and hepatic dysfunction was identified as alanine aminotransferase or aspartate aminotransferase amounts twice or more than the normal value. Meanwhile, the ankle–brachial index (ABI) was identified by the ratio of SBP recorded at the ankle to SBP obtained at the brachial artery. The absolute difference in SBP between the right and left brachial arteries is called interarm blood pressure differential (IAD). Smoking and drinking habits were self‐reported and categorized as never smokers/drinkers, past smokers/drinkers, or current smokers/drinkers. Physical activity was defined as engaging in any exercise for at least 30 minutes three or more times a week.

### 2.3 Measurement of arterial stiffness

Between January 2010 and December 2016, baPWV was performed to assess arterial stiffness using the web‐based arterial stiffness detection system BP‐203 RPE III (Omron Health Healthcare Co., LTD.).^15^ Qualified nurses conducted the procedure between 7 and 9 am. The participants were required to sit in a room controlled at 22**–**25°C for at least 30 minutes. Subjects were requested to lie supine and stay quiet during the assessment. Then we placed blood pressure cuffs on their arms and ankles. While the lower malleolus cuff's bottom edge was 1–2 cm just above the medial malleolus, the inferior margin of the cuff was 2–3 cm just above the fossa cubitus. A cardiac sound monitor was positioned on the left side of the sternum. The assessment was performed twice separately, with the second measurement considered the final outcome. As per the previous study, BaPWV <1400 cm/s was considered ideal vascular function, whereas 1400 ≤baPWV <1800 cm/s was considered as borderline arterial stiffness, and baPWV ≥1800 cm/s was indicated high‐grade arterial stiffness.^15^

### 2.4 Outcome assessment

All cancers were confirmed from hospital diagnostic data depending on the international classification of diseases‐10 (ICD‐10) code. These include cancers of the digestive system (C15–C26), respiratory system (C34 + C39), genitourinary system (C50–C58 in women and C60–C63 in men), and other system cancers (all with codes beginning with C). During follow‐up, hospitalization records were utilized to identify deaths. We also screen death certificates from provincial unit demographic offices annually to improve the quality of our data on outcome events. All patients were monitored until the ACMC or December 31, 2018, whichever happened first.

### 2.5 Statistical analyses

For the baseline description, the normally distributed variables were described as mean ± standard deviation (SD). ANOVA was employed to investigate the comparison between three or more groups. The categorical variables were represented as counts and percentages (%), and the chi‐square test was used to investigate the comparison between groups. The cumulative incidence of ACMC in different baPWV cohorts was compared by the Kaplan–Meier survival curves. To account for potential confounding effects, we selected variables with *p* < 0.05 in the univariate Cox analyses or clinically associated with cancer occurrence as candidates for the multivariate Cox regression analysis. We performed bootstrapping procedure in the framework of the Cox regression model to investigate stability and to select factors included in a final model. The proportional hazard assumption in the Cox model was tested and satisfied in all cases using the Schoenfeld residuals (PH hypothesis), which was used to investigate whether the impact of different baPWV levels on ACMC changed over time. To exclude the influence of age, sex, blood flow dynamics (represented by MAP), nutritional status (represented by BMI), metabolic diseases (represented by DM, HDL, and high levels of uric acid), chronic inflammation (represented by hs‐CRP levels), lifestyle behaviors (represented by smoking, drinking, exercise), and hepatitis B (represented by HBsAg positive history), liver and kidney function, and family cancer history, we employed multivariate Cox proportional hazards regression model to analyze the impact of various baPWV cohorts on ACMC. We further determined the spline curve of baPWV on ACMC to explore whether a positive relationship existed between baPWV and ACMC. The Cox model was repeated after age and sex stratification to inspect the influence of baPWV on malignancy mortality in different age cohorts and sex cohorts. Participants with ABI ≤0.9 and/or IAD ≥15 mmHg at baseline were excluded from the sub‐group analysis to exclude the confounding effects of peripheral vascular disease on arterial stenosis. *p* < 0.05 (two‐sided test) was considered statistically significant.

## 3 RESULTS

### 3.1 Descriptive analysis

From January 1, 2010 to December 31, 2018, 43,943 individuals were followed up for the endpoint of ACMC. During a total follow‐up duration of 172,775.69 person‐years, we detected 553 new cancer cases. Of them, 157 died. The baseline data show that the average age was 47.84 ± 12.74 years old, and 31,554 (71.81%) were male. Participants with higher baPWV had higher age, SBP, DBP, MAP, BMI, TC, HDL, FPG, UA, and eGFR. In addition, the participants with higher baPWV had a higher proportion of HTN, DM, current drinkers, current smokers, physical activity, and hepatic dysfunction (all *p* < 0.05) than those with lower baPWV. Table 1 illustrates the comparison of the initial features of different cohorts.

**TABLE 1:** Baseline clinical characteristics of participants.

Variables	baPWV <14.0 m/s (*n* = 18,911)	14.0 ≤ baPWV <18.0 m/s (*n* = 17,141)	baPWV ≥18.0 m/s (*n* = 7891)	*p‐*Value
Age (year)	41.36 ± 9.68	49.40 ± 10.24	60.02 ± 10.98	<0.001
Male, *n* (%)	11,081 (58.59)	14,004 (81.70)	6469 (81.99)	<0.001
SBP (mmHg)	120.81 ± 13.58	135.02 ± 16.44	147.65 ± 19.44	<0.001
DBP (mmHg)	77.23 ± 9.51	84.22 ± 10.21	87.11 ± 11.42	<0.001
MAP (mmHg)	91.73 ± 10.10	101.42 ± 11.04	106.04 ± 11.28	<0.001
BMI (kg/m^2^)	24.31 ± 3.19	24.98 ± 3.00	25.26 ± 3.02	<0.001
hs‐CRP >2 mg/L (%)	3986 (21.07)	4779 (27.88)	2628(33.30)	<0.001
TC (mmol/L)	4.57 ± 1.35	4.99 ± 1.46	5.14 ± 1.92	<0.001
HDL (mmol/L)	1.44 ± 0.67	1.44 ± 0.68	1.48 ± 0.72	<0.001
FPG (mmol/L)	5.25 ± 1.04	5.66 ± 1.64	6.58 ± 2.15	<0.001
Uric acid (mmol/L)	302.05 ± 95.40	316.75 ± 97.15	327.01 ± 95.61	<0.001
eGFR (mL/minute × 1.73 m^2^)	101.44 ± 22.60	96.76 ± 21.50	87.26 ± 20.35	<0.001
Current smoker, *n* (%)	5106 (27.01)	6028 (35.17)	2266 (28.72)	<0.001
Current drinker, *n* (%)	571 (3.02)	953 (5.56)	466 (5.91)	<0.001
Physical activity, *n* (%)	9792 (51.78)	9071 (52.92)	4250 (53.86)	0.011
Hepatic dysfunction, *n* (%)	1597 (8.44)	1930 (11.26)	699 (8.86)	<0.001
Anemia, *n* (%)	1244 (6.58)	781 (4.56)	304 (3.86)	<0.001
HBsAg positive, *n* (%)	173 (0.92)	143 (0.83)	59 (0.75)	0.262
Antihypertensive use, *n* (%)	698 (3.69)	2581 (15.06)	2342 (29.68)	<0.001
Tumor family history, *n* (%)	527 (2.79)	786 (4.59)	622 (7.89)	<0.001

Abbreviations: baPWV, brachial–ankle pulse wave velocity; BMI, body mass index; DBP, diastolic blood pressure; eGFR, estimated glomerular filtration rate; FPG, fasting plasma glucose; HBsAg, hepatitis B surface antigen; HDL‐C, high‐density lipoprotein cholesterol; hs‐CRP, high sensitivity C‐reactive protein; MAP, mean arterial pressure; SBP, systolic blood pressure; TC, total cholesterol.

### 3.2 All‐cause mortality after cancer

**FIGURE 2:**
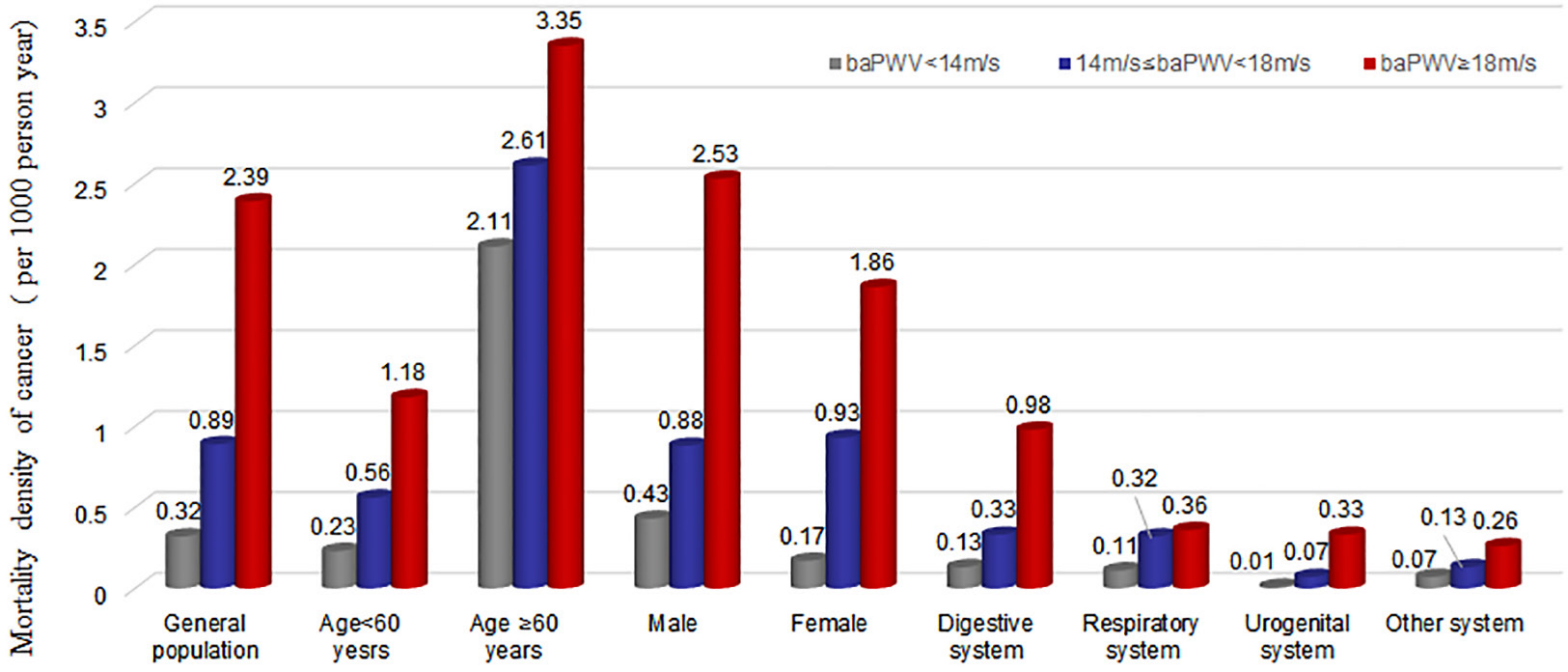
Incidence density of cancer mortality (per 1000‐person year) in different gender, age groups and different systems. Incidence density of cancer mortality in the general population, individuals <60 years old, and individuals ≥60 years old, male, female, and subgroups of different systems: digestive system, respiratory system and urogenital systems, and other systems.

There were 157 ACMC (digestive system: 62; respiratory system: 57 cases; urogenital system: 16 cases; other systems: 22 cases) in 153,889.58 person‐years follow‐up time. Figure 2 presents the cumulative ACMC in different baPWV cohorts. ACMC increased with PWV in different age groups and sex cohorts (*p* < 0.05). We observed significant variations in ACMC in the entire population, including in the digestive, respiratory, and urogenital systems. However, ACMC has no significant variations across the different baPWV groups in the “other systems.”

### 3.3 Risk of ACMC according to the baPWV

The cumulative occurrence of ACMC revealed an increasingly greater risk across normal to progressive arterial stiffness cohorts. We observed a significant relationship between arterial stiffness severity and malignancy mortality (hazard ratio [HR] = 2.72, 95% confidence interval [CI]: 1.55–4.80; *p* < 0.001) among those with baPWV ≥18 m/s (Table 2).

**TABLE 2:** Risk of ACMC according to baPWV.

PWV (m/s)	ACMC	
	HR (95% CI)	*p* for trend
Entire cohort (*n* = 43,943)		
<14.0	Ref.	0.0003
14.0–18.0	1.67 (1.00, 2.79)	
≥18.0	2.72 (1.55, 4.80)	
per 1 SD increase	1.20 (1.03, 1.41)	0.021
Age < 60 years (*n* = 35,512)		
<14.0	Ref.	0.002
14.0–18.0	1.94 (1.02, 3.69)	
≥18.0	3.51 (1.56, 7.90)	
per 1 SD increase	1.44 (1.10, 1.89)	0.008
Age ≥60 years (*n* = 8431)		
<14.0	Ref.	0.056
14.0–18.0	0.96 (0.42, 2.20)	
≥18.0	1.55 (0.69, 3.51)	
per 1 SD increase	1.10 (0.90, 1.33)	0.356
Men (*n* = 31,554)		
<14.0	Ref.	0.001
14.0–18.0	1.43 (0.81, 2.53)	
≥18.0	2.65 (1.43, 4.92)	
per 1 SD increase	1.20 (1.01, 1.43)	0.042
Women (*n* = 12,389)		
<14.0	Ref.	0.316
14.0–18.0	2.32 (0.78, 6.94)	
≥18.0	2.24 (0.59, 8.52)	
per 1 SD increase	1.16 (0.80, 1.70)	0.435

*Note*: Adjusted for age and sex, MAP, body mass index, hs‐CRP, total cholesterol, fasting plasma glucose, uric acid, eGFR, current smoker, current drinker, high/intensive activity, hepatic dysfunction, anemia, antihypertensive use, HBsAg positive, and tumor family history. 1 SD of baPWV: 3.52 m/s.

Abbreviations: ACMC, all‐cause mortality in cancer patients; baPWV, brachial–ankle pulse wave velocity.

In contrast to males, we observed a non‐significant relationship between the measures of baPWV severity and ACMC in females (all *p* > 0.05). We observed HR of 1.20 (95% CI: 1.03–1.41; *p* < 0.05) per 1‐SD increase in baPWV among the entire cohort. Likewise, the HR of 1.44 (95% CI: 1.10–1.44; *p* = 0.008) and 1.20 (95% CI: 1.01–1.43; *p* < 0.05) in those less than 60 years old and men population (Table 2). Participants with increased baPWV were more likely to be diagnosed with ACMC in digestive cancer and the respiratory system (Table 3).

**TABLE 3:** Adjusted hazard ratio (HR) associated with ACMC of different systems.

PWV (m/s)	ACMC	
	HR (95% CI)	*p* for trend
Digestive system (*n* = 62)		
<14.0	Ref.	0.017
14.0–18.0	1.37 (0.62, 3.05)	
≥18.0	2.61 (1.09, 6.24)	
per 1 SD increase	1.26 (0.99, 1.61)	0.061
Respiratory system (*n* = 57)		
<14.0	Ref.	0.011
14.0–18.0	2.23 (0.94, 5.25)	
≥18.0	3.47 (1.33, 9.06)	
per 1 SD increase	1.26 (0.97, 1.63)	0.089
Urogenital system (*n* = 16)		
<14.0	Ref.	0.123
14.0–18.0	2.63 (0.28, 25.11)	
≥18.0	5.05 (0.49, 52.24)	
per 1 SD increase	0.92 (0.55, 1.55)	0.754
Other systems (*n* = 22)		
<14.0	Ref.	0.757
14.0–18.0	1.22 (0.36, 4.20)	
≥18.0	1.28 (0.30, 5.52)	
per 1 SD increase	1.15 (0.74, 1.79)	0.538

*Note*: Adjusted for age and sex, MAP, body mass index, hs‐CRP, total cholesterol, fasting plasma glucose, uric acid, eGFR, current smoker, current drinker, high/intensive activity, hepatic dysfunction, anemia, antihypertensive use, HBsAg positive, and tumor family history. 1 SD of baPWV: 3.52 m/s.

Abbreviations: ACMC, all‐cause mortality in cancer patients; baPWV, brachial–ankle pulse wave velocity.

**FIGURE 3:**
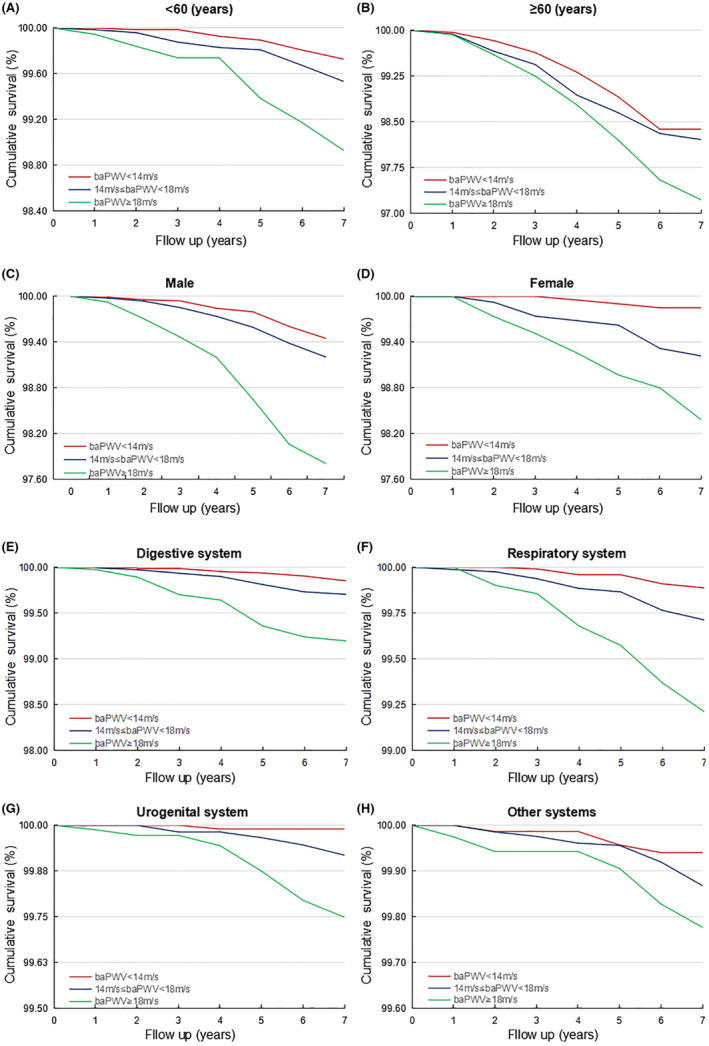
The Kaplan–Meier survival curves for cancer mortality. The Kaplan–Meier survival curves for cancer mortality in the participants ˂60 years (A), participants ≥60 years male (B), males (C), female (D), digestive system (E), respiratory system (F), urogenital system (G), and other systems (H).

**FIGURE 4:**
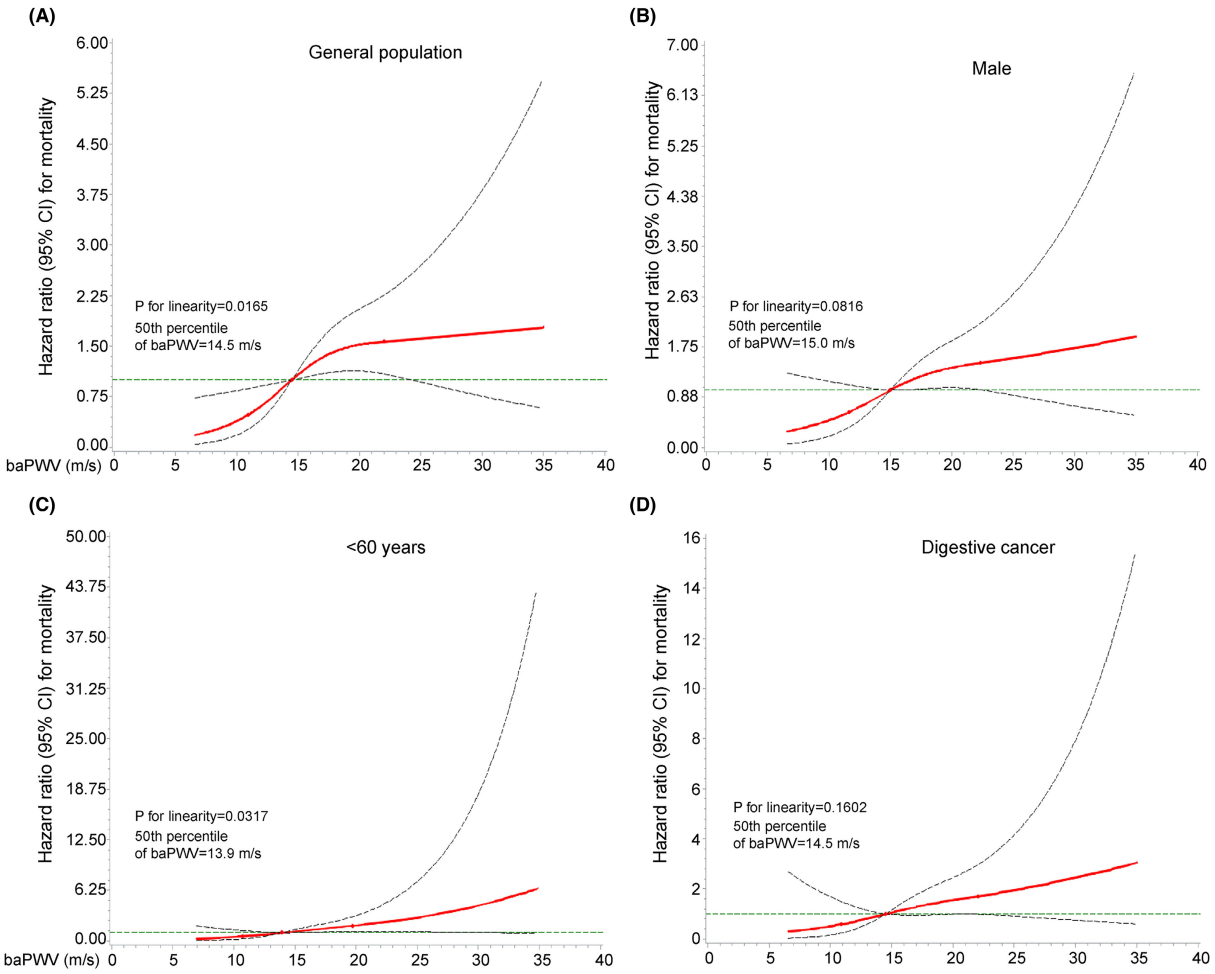
The risk of cancer mortality according to restricted spline curve analysis. Spline‐curve analyses show a linearly increasing risk, linking the severity of arterial stiffness to overall cancer mortality risk in the general population (A), men (B), participants <60 years old (C), and individuals with digestive cancer (D).

Figure 3 describes the risk of ACMC according to Kaplan‐Meir analysis. There was significant difference in the proportion of ACMC in the different groups of baPWV in the entire cohort (*χ* ^2^ = 100.86, *p* < 0.001), men (*χ* ^2^ = 58.43, *p* < 0.001), participants ˂60 years (*χ* ^2^ = 24.05, *p* < 0.001), and those with cancer of digestive system (*χ* ^2^ = 44.66, *p* < 0.001). The Schoenfeld residuals analysis confirmed no linear association between Schoenfeld residuals and time rank, as demonstrated by the corresponding *p*‐value of Pearson correlation (Figure S1). Interestingly, when baPWV (restricted cubic spline, section 3 at 5%, 50%, and 95%) was calculated, the observed correlation with high baPWV was unchanged. Spline analysis showed a linearly increased risk of arterial stiffness with overall ACMC risk in the entire cohort (*p* = 0.0165) and other cohorts, including men (*p* = 0.0816), participants <60 years of age (*p* = 0.0317), and digestive cancer patients (*p* for trend = 0.1602) (Figure 4).

### 3.4 Subgroup analyses

**FIGURE 5:**
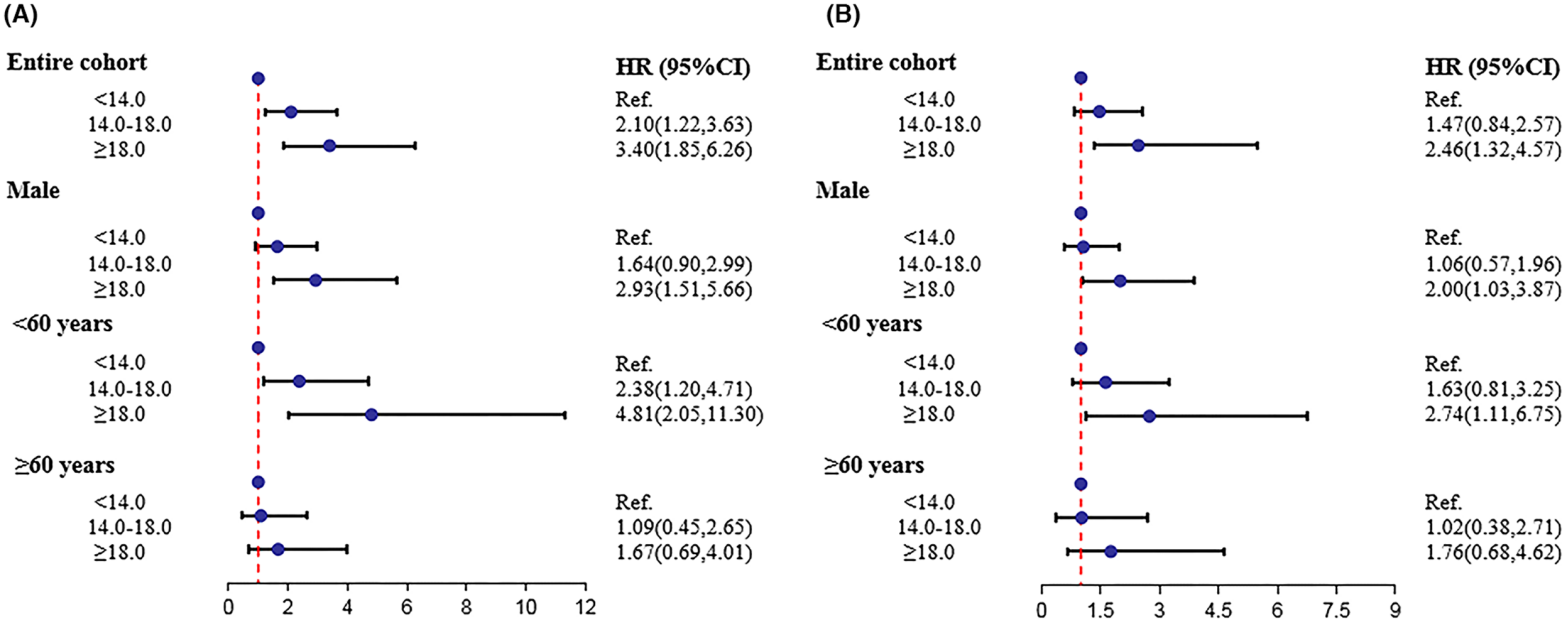
Sensitivity analyses for cancer mortality. The hazard ratio and 95% CI for cancer mortality after cancer in the general participants without peripheral vascular diseases (A) and (B) the population excluding participants who died within the first year of follow‐up.

To validate the impact of baPWV level on ACMC, we further evaluated the method in participants without peripheral vascular diseases. After excluding participants who died within 1 year of follow‐up, the Cox model was repeated for sub‐group analysis. The results showed that baPWV was statistically significant in the 60‐year‐old and male populations (Figure 5).

## 4 DISCUSSION

The results of this large‐scale observation study unveiled a close association between arterial stiffness and ACMC. Furthermore, our study found that high baPWV was positively associated with an increased risk of subsequent all‐cause mortality.

In the past, several studies demonstrated an association between arterial stiffness and CVD,^16^ DM,^17^ and renal disease^18^ during/after chemotherapy. In a cohort study from the Kailuan Community, Zhou et al. discovered a greater risk of clinical adverse events in normotensive and high blood pressure cohorts with raised arterial stiffness compared to those without arterial stenosis (ideal vascular function cohort).^19^ This implies that the presence of arterial stenosis, despite the blood pressure status, may determine the risk of all‐cause mortality. Besides, Vlachopoulos et al. reported a meta‐analysis of 17 longitudinal studies to assess the association between aorta PWV and all‐cause of death among 15,877 patients. The authors concluded that each rise in aortic PWV of 1 m/s was related to a 15% higher risk of all‐cause death.^20^ Indeed, their finding provides insight into the relationship between aortic stenosis and adverse clinical outcomes. However, their study left the question of whether PWV measured from other body parts associated with all‐cause mortality open. As such, there was a lack of robust knowledge on whether arterial stiffness measured by baPWV is associated with ACMC. To our knowledge, this was the first study to evaluate the association between baPWV and ACMC.

The present study shows that increased arterial stiffness, as measured by baPWV, was associated with all‐cause mortality. Importantly, we observed an increased risk of all‐cause mortality with an increase in 1 SD in baPWV among the participants in the general population. However, the association between arterial stiffness and all‐cause mortality events was found to differ in sex. The high baPWV was increasingly associated with a risk of all‐cause mortality in men after adjusting for potential confounders, but not in women. This discrepancy could be explained by the difference in the prevalence of HTN between men and women, which is higher in men,^21^ and its associated adverse cardiovascular outcomes.^22^ In addition, a previous study reported a higher risk of cancer in the HTN group than non‐HTN patients.^23^ Furthermore, Jessica et al. found that subjects with SBP 160–179 mmHg or DBP 100–10 mmHg had an 8 and 36% increased risk for cancer incidence and mortality, respectively, compared with the lowest grade of HTN.^24^

In our study, baPWV was significantly associated with ACMC in men and participants younger than 60 years of age. Although the risk trend for baPWV and ACMC increased in those >60 years old and women, no statistically meaningful association was noticed after multivariate analysis. As per our result, age and gender could affect baPWV measurement, and it should be studied and interpreted with caution. Thus, further studies are required to understand the effect of age and gender‐related physiological changes that affect baPWV. In the past, baPWV was significantly associated with subclinical target organ damage in participants younger than 65 years.^25^ The baPWV is known to increase with aging synchronously.^14^, ^26^ Among the vascular and metabolic risk factors, age and SBP are the strongest determinants of baPWV.^27^ According to the study of Yu et al.^28^, age and SBP also showed stronger predictors than other vascular risk factors in Chinese. Meanwhile, the age‐dependent baPWV differed in sexes, arterial stiffness were observed in men from adolescence to 58 years, and in women thereafter. However, the Kailuan Community Cohort recruited study participants from a coal company, with an average age of 47.84 ± 12.74 years old, and 31,554 (71.81%) were male. Thus, we suggest a further follow‐up study with careful sampling that considers age and gender‐based analysis to validate the potential benefit of baPWV screening in the middle‐aged population.

PWV increases with the risk of cardiovascular disease.^29^ However, the present study's results remain unchanged after excluding the effects of severe vascular diseases. The data strongly indicates the presence of an association between arterial stiffness and all‐cause mortality. This implies, in the future, the possibility of defining risk thresholds using baPWV, specifically tailored to the contemporary risk stratification concept of onco‐cardiology. Also, our results may indicate the importance of baPWV to improve the concept of risk stratification along with already established grading algorithms.

When arterial stiffness occurs, the arterial system undergoes complicated structural and functional alterations. Several processes may cause the correlation between arterial stiffness and the risk of dying from malignancy. First, endothelial injury followed by intimal thickening and the increased permeability due to intimal thickening may represent the development of arterial stiffness.^30^ In primary cancer, an endothelial injury that results in increased endothelial permeability leads to the effective extravasation of cancer cells at metastatic sites.^31^ Second, oxidative stress and increased inflammation may be the main risk factors for arterial stiffness and ACMC.^32^ Moreover, microRNA expression patterns and autophagy contribute to progressive increases in arterial stiffness.^30^ miRNA (first microRNA) gene dysregulation is a common cancer‐causing factor, and miRNAs may either inhibit cancer or serve as oncogenes.^33^ Strategies to promote and suppress autophagy have been suggested as a cancer treatment because autophagy has conflicting, context‐dependent functions in malignancy.^34^, ^35^

To the extent of our knowledge, this is the first major study to recognize the relationship between baPWV and ACMC. However, this study must be interpreted in the context of some significant limitations. Assessing arterial stiffness as a marker of medial layer vascular function is an operator‐dependent procedure. We do not consider carotid–femoral pulse wave velocity (c‐fPWV), the gold standard measurement for arterial stenosis, to test the association between arterial stenosis and all‐cause mortality. Therefore, we failed to validate the observed relationship between baPWV and all‐cause of death by replicating the analysis using c‐fPWV. Also, our study only registered the Chinese population living in Northern China, which may limit the generalization of our results. However, the homogeneous nature of our prospective study may provide absolute control of potential confounding effects that could result from racial disparities and health service inequalities. The Kailuan community cohort recruits study participants from a coal company. Therefore, more males than females were included in the study. Though the enrolment of only Chinese ethnicity in this study could bolster its internal validity, further research is needed to confirm whether this association exists among other ethnic groups.

## 5 CONCLUSION

In conclusion, the results of this study demonstrate a positive association between high baPWV and ACMC, with a potentially novel correlation. The association between high baPWV (≥18 m/s) and risk of all‐cause mortality was prominent in men and those ≤60 years of age. Future studies must clarify whether interventions that decrease arterial stiffness reduce or delay deaths among cancer patients. This study provides insight into the benefit of preventing arterial stenosis.

## AUTHOR CONTRIBUTIONS

**Jiatian Li:** Conceptualization (equal); methodology (equal); software (equal). **Tesfaldet Habtemariam Hidru:** Data curation (equal); writing – original draft (equal). **Yajuan Lin:** Investigation (lead); validation (lead). **Xinying Wang:** Investigation (equal); validation (equal). **Li Lin:** Investigation (lead); validation (lead). **Shuohua Chen:** Supervision (equal). **Yunlong Xia:** Software (equal); validation (equal). **Xiaolei Yang:** Conceptualization (equal); methodology (equal); software (equal). **Shouling Wu:** Project administration (equal); writing – review and editing (equal).

## FUNDING INFORMATION

This work was supported by the Chang Jiang Scholars Program (T2017124 to YX); the National Natural Science Foundation of China (81900439 to XY), and Liao Ning Revitalization Talents (XLYC2002096 to YX); the Science Foundation of Doctors of Liaoning Province (2020‐BS‐197 to XY).

## CONFLICT OF INTEREST STATEMENT

The authors declare that the research was conducted in the absence of any commercial or financial relationships that could be construed as a potential conflict of interest.

## ETHICS STATEMENT

Each individual signed a written agreement statement and consented to use the information for analysis. The Kailuan General Hospital Ethics Committee authorized the investigation, ensuring the study procedure complied with the Declaration of Helsinki II requirements.

## CLINICAL TRIAL REGISTRATION

Clinical trial number ChiCTR‐TNRC‐11001489.

## CONTRIBUTION TO THE FIELD STATEMENT

According to World Health Organization (WHO), cancer is a leading cause of death worldwide. In parallel, the crude incidence and mortality rates of cancer have progressively risen since 2000 in China. According to the most recent epidemiological and statistical information, China is expected to have 3,210,000 cancer deaths and 4,820,000 new cases of malignancy in 2022. Therefore, there is an urgent need to discover risk factors influencing cancer mortality initiation and progression. Arterial stiffness is a well‐established indicator of the rigidity of the artery wall. It can be determined by a simple and noninvasive procedure known as the brachial–ankle pulse wave velocity (baPWV). Of note, baPWV is defined as the distance between the brachial and the tibial artery divided by the transit time between these two arteries. The relationship between arterial stiffness and deaths in people with Type 1 diabetes and hypertension has been investigated in the past. Similarly, a community‐based study showed that arterial stiffness was positively associated with all‐cause mortality in elderly Japanese individuals. Therefore, the purpose of this study was to investigate the association between arterial stiffness status measured by baPWV and all‐cause mortality in Chinese cancer patients.

## Supporting information


Figure S1.

## Data Availability

The data used to support the findings of this study are included within the article.
