# Supplementary material for: Arterial stiffness is associated with cancer mortality: Insight from Kailuan study
Source: Cancer Med. 2023 Jun 23;12(15):16580–90. doi: 10.1002/cam4.6251 (PMC10469642; doi:10.1002/cam4.6251)
Supplement: Supplementary file 1 — Figure S1. [file CAM4-12-16580-s001.docx]

**
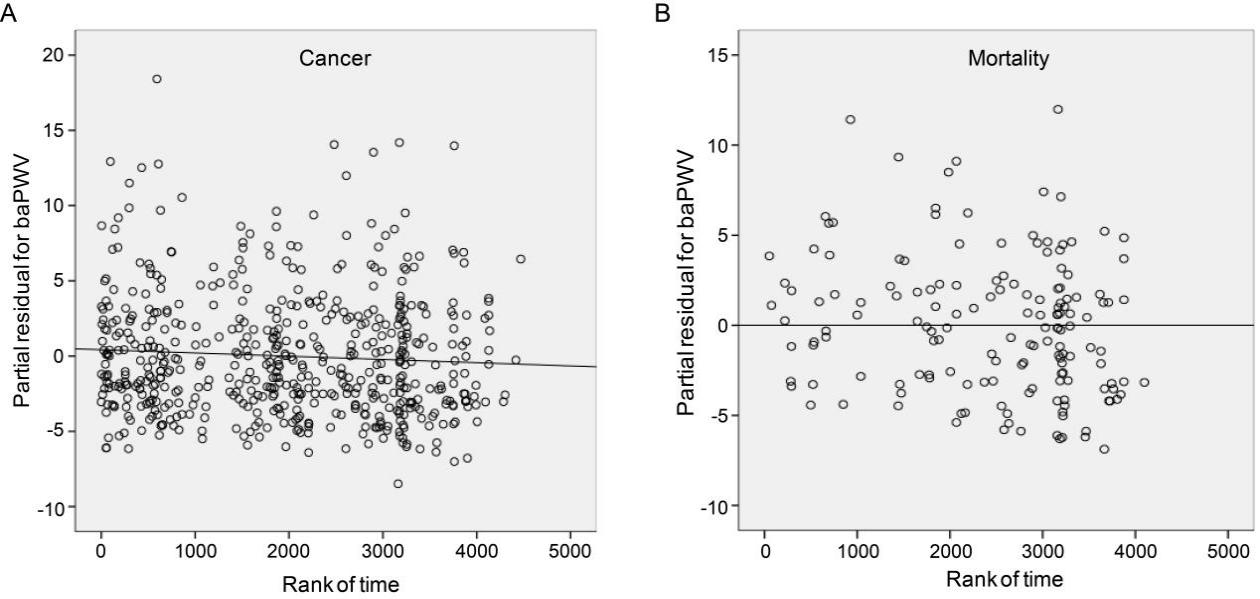
**

**Supplemental Figure 1.** **The smoothing curve of Schoenfeld partial residuals against time rank.**

The smoothing curve of cancer mortality in all participants.
